# Scaling parameter of the lethal effect of mammalian cells based on radiation-induced OH radicals: effectiveness of direct action in radiation therapy

**DOI:** 10.1093/jrr/rraa111

**Published:** 2020-12-14

**Authors:** Tamon Kusumoto, Ryo Ogawara, Kazuyo Igawa, Kentaro Baba, Teruaki Konishi, Yoshiya Furusawa, Satoshi Kodaira

**Affiliations:** 1 National Institutes for Quantum and Radiological Science and Technology, 4-9-1 Anagawa, Inage-ku, 263-8555 Chiba, Japan; 2 Advanced Research Center for Beam Science, Institute for Chemical Research, Kyoto University, Gokasho, Uji, Kyoto 611-0011, Japan; 3 Neutron Therapy Research Center, Okayama University, 2-5-1 Shikata, Kita-ku, 700-8558 Okayama, Japan; 4 Graduate School of Biomedical Science and Engineering, Hokkaido University, Kita-12 Nishi-5, Kita-ku, 080-0808 Hokkaido, Japan

**Keywords:** OH radicals, V79 cell, surviving fraction, direct action, indirect action

## Abstract

We have been studying the effectiveness of direct action, which induces clustered DNA damage leading to cell killing, relative to indirect action. Here a new criterion Direct Ation-Based Biological Effectiveness (DABBLE) is proposed to understand the contribution of direct action for cell killing induced by C ions. DABBLE is defined as the ratio of direct action to indirect action. To derive this ratio, we describe survival curves of mammalian cells as a function of the number of OH radicals produced 1 ps and 100 ns after irradiation, instead of the absorbed dose. By comparing values on the vertical axis of the survival curves at a certain number of OH radicals produced, we successfully discriminate the contribution of direct action induced by C ions from that of indirect action. DABBLE increases monotonically with increasing linear energy transfer (LET) up to 140 keV/μm and then drops, when the survival curves are described by the number of OH radicals 1 ps after irradiation. The trend of DABBLE is in agreement with that of relative biological effectiveness (RBE) of indirect action. In comparison, the value of DABBLE increases monotonically with LET, when the survival curves are described by the number of OH radicals 100 ns after irradiation. This finding implies that the effectiveness of C ion therapy for cancer depends on the contribution of direct action and we can follow the contribution of direct action over time in the chemical phase.

## INTRODUCTION

The number of patients receiving radiation cancer treatments is increasing annually [1]. To understand therapeutic effects and to minimize medical exposures, many researchers have examined surviving fractions not only of cancer tissues [2, 3] but also of normal tissues [4] irradiated with photons, protons and heavy ions. Usually, relative biological effectiveness (RBE) is defined for survival at a given level (often 10% survival) for a certain radiation relative to that for ^60^Co gamma rays or X-rays [5–9]. One of the concerns of RBE is that the radiation quality (i.e. difference in type of ionizing radiation) is not properly considered when the absorbed dose is applied in description of the survival curves, namely the absorbed dose might not be a universal parameter. That means that we cannot take into account the difference between a local energy deposition by incoming ions and spatially homogeneous ones by low linear energy transfer (LET) radiations (i.e. gamma rays and X-rays). In other words, the lethal effect induced by incoming ions would differ depending on whether ions hit the cell nucleus or not, even at the same absorbed dose [10–12]. Therefore, the survival curves of ion beams expressed by absorbed dose cannot be simply compared to those of low LET radiations. A new parameter replacing RBE is required for expressing the biological effectiveness universally, while considering the difference in radiation type.

**Fig. 1.**
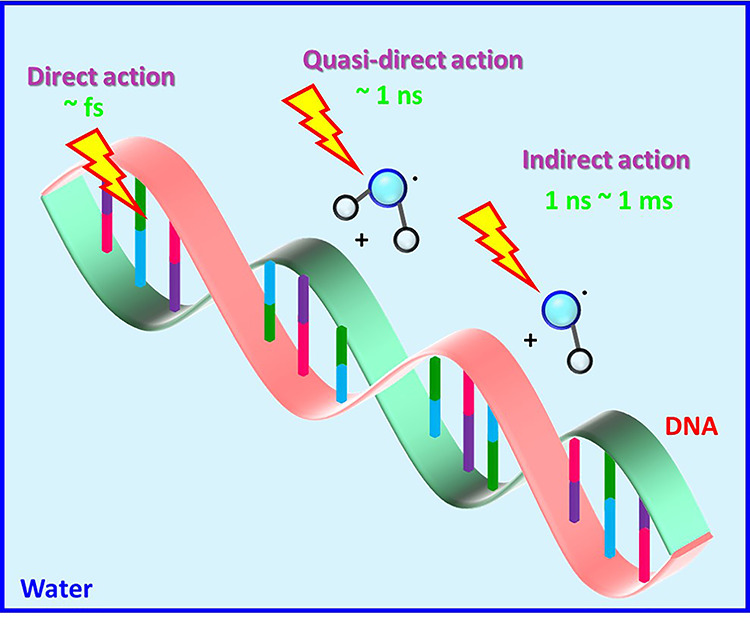
A schematic picture of direct, quasi-direct and indirect actions. Direct action is caused by direct ionization or excitation of DNA in the physical phase on a fs timescale. Indirect action occurs by the reaction of DNA molecules with water radiolysis products in the chemical phase. Furthermore, quasi-direct action, which is DNA damage induced by ionization of tightly bound water molecules of the primary hydration layer, also contributes to DNA damage leading to cell killing in the early chemical phase (~1 ns).

Previously, the validity of the use of the number of OH radicals produced as a universal criterion for expressing the biological effectiveness was shown in an accelerator-based neutron field [13, 14]. ^60^Co gamma ray equivalent doses of thermal neutrons, fast neutrons and contamination gamma rays were successfully evaluated while considering the difference in the radiation quality. ^60^Co gamma ray equivalent dose is known as a standard for discussing the biological effectiveness induced by the ionizing radiations. Thus, the evaluation of ^60^Co equivalent dose from the number of OH radicals produced is important to compare the biological effectiveness between radiations with different radiation qualities. In the present study, we first apply the number of OH radicals produced as a universal parameter to discuss the effectiveness of C ion therapy for cancer. Among water radiolysis products, OH radicals react more efficiently with proteins, DNA and many small molecules in cells (e.g. thiols and amino acids) relative to other water radiolysis products (Fig. 1) [15]. Comparison of the surviving fractions due to different radiations at the same number of OH radicals produced is relevant to investigating the contribution of the direct-type action, which is likely to induce clustered DNA damage associated with cell killing [16]. We note that the direct-type action includes not only direct ionization or excitation of DNA but also ionization of water molecules of solvation followed by hole and electron transfers to the DNA as shown in Fig. 1. Indeed, changes in DNA conformation and the attack of water radiolysis products from bulk water on DNA play an important role in radiation-induced DNA base damage (Fig. 1) [17]. Therefore, such reactions are considered as part of direct action, so called quasi-direct action, because of its time scale (~1 ns) [18]. Water radiolysis products contribute not only to indirect action but also direct action. From this point of view, we describe survival curves by the number of OH radicals produced and then propose a new concept Direct Action-Based BiologicaL Effectiveness (DABBLE) to discuss the contribution of direct action.

## METHODS

### Monte Carlo simulation in Geant4-DNA

A Monte Carlo simulation in the Geant4-DNA toolkit was carried out to estimate the number of OH radicals produced by C ions with energies from 5 MeV to 5 GeV [19–21]. We used the ‘G4EmDNAPhysics_option8’ physics constructor in Geant4 version 10.05.p01, in which the ionization processes of protons and heavy ions (B, Li, C, N, O, Si and Fe) were installed [22]. This physics constructor covers the energy range from 0.5 to 10^6^ MeV/u for heavy ions. Additionally, we used the ‘G4EmDNA Chemistry’ chemical constructor, in which yields of water radiolysis products (e.g. OH radicals, hydrated electrons and hydrogen peroxide) could be calculated accurately [23]. The simulation geometry consisted of a 10 × 10 × 10 mm^3^ air-free water cube. When primary particles (i.e. C ions) deposited 10 keV of their energies into the water cube, the tracking of the physical process ceased and shifted to the chemical process. We aborted the simulation when the total energy deposition of each event was >10.1 keV, meaning that the total energy deposition of each event was always between 10 and 10.1 keV. In the present study, the number of OH radicals produced was simulated at 1 ps and 100 ns after the ion pass. The former is the beginning of the chemical phase and the latter is the middle of the chemical phase in which radical–radical reactions subside.

### Derivation of fitting curves of indirect action by X-rays

To derive DABBLE, we have to discriminate the surviving fraction induced by indirect action from that by direct action. In general, the survival curve is described as a function of absorbed dose *D* (Gy) using a linear quadratic (LQ) model, which is the theory describing the survival curve based on dual action [24], as,

${\mathrm{SF}}_{\mathrm{t}}(D)=\exp \big\{-\big({\alpha}_DD+{\beta}_D{D}^2\big)\big\}$ (1)

where SF_t_(*D*) is a total (direct action + indirect action) surviving fraction described as a function of absorbed dose, α*_D_* (/Gy) is a fitting parameter referring to the linear term and β*_D_* (/Gy^2^) is a fitting parameter referring to the quadratic term. The number of OH radicals produced *N_OH_* (/L) is estimated from the G value and absorbed dose as,

(2) \begin{equation*} {\mathrm{N}}_{\mathrm{OH}}=\frac{\mathrm{G}\left(\mathrm{OH}\right)}{100}\times \frac{\mathrm{D}}{\mathrm{e}} \end{equation*}

where G(*N_OH_*) is the G value of OH radicals produced and e is the elementary charge (1.6 × 10^−19^ C). Then, the surviving fraction is expressed as a function of *N_OH_*:

(3) \begin{equation*} {\mathrm{SF}}_{\mathrm{t}}\left({\mathrm{N}}_{\mathrm{OH}}\right)=\exp \left[-\left\{{\alpha}_{\mathrm{OH}}{\mathrm{N}}_{\mathrm{OH}}+{\beta}_{\mathrm{OH}}{{\mathrm{N}}_{\mathrm{OH}}}^2\right\}\right] \end{equation*}

where α_OH_ and β_OH_ are fitting parameters. When the surviving fraction is 0.1, 90% of the cells are dead. So, the death fraction (not surviving fraction) by indirect action *DF_in_* is written as,

(4) \begin{equation*} {\mathrm{DF}}_{\mathrm{in}}\left({\mathrm{N}}_{\mathrm{OH}}\right)=k\times \left\{1-{\mathrm{SF}}_{\mathrm{t}}\left({\mathrm{N}}_{\mathrm{OH}}\right)\right\} \end{equation*}

where *k* is a previously obtained contribution ratio of indirect action (the number of cells killed by indirect action/the number of cells killed). A lot of researchers have studied the contribution of indirect action [25–28]. Notably, Hirayama *et al*. reported that *k* of 200 kVp X-rays for lethal effects of V79 cells was 0.76 [28]. So, the surviving fraction of indirect action SF_in_(*N_OH_*) is simply expressed as,

(5) \begin{eqnarray*} {\mathrm{SF}}_{\mathrm{in}}\left({N}_{OH}\right) &=& 1-{\mathrm{DF}}_{\mathrm{in}}\left({N}_{OH}\right) \\ &=& 1-k\times \left\{1-{\mathrm{SF}}_{\mathrm{t}}\left({N}_{OH}\right)\right\}\nonumber \\ &=&k\times{\mathrm{SF}}_{\mathrm{t}}\left({N}_{OH}\right)+\left(1-k\right)\nonumber \end{eqnarray*}

From equations (3) and (5), SF_in_(*D*) is written as,

(6) \begin{equation*} {\mathrm{SF}}_{\mathrm{in}}\left({N}_{OH}\right)=k\times \exp \left[-\left\{\alpha{N}_{OH}+\beta{N_{OH}}^2\right\}\right]+\left(1-k\right) \end{equation*}

In the same way, the surviving fraction of direct action SF*_d_* is written as,

(7) \begin{eqnarray*} {\mathrm{SF}}_{\mathrm{d}}\left({N}_{OH}\right) &=& 1-{\mathrm{DF}}_{\mathrm{d}}\left({N}_{OH}\right) \nonumber\\ &=& 1-\left(1-k\right)\times \left\{1-{\mathrm{SF}}_{\mathrm{t}}\left({N}_{OH}\right)\right\} \nonumber\\ &=& \left(1-k\right)\times{\mathrm{SF}}_{\mathrm{t}}\left({N}_{OH}\right)+k \nonumber\\ &=& \left(1-k\right)\times \exp \left[-\left\{\alpha{N}_{OH}+\beta{N_{OH}}^2\right\}\right]+k \end{eqnarray*}

## RESULTS AND DISCUSSION

### G values of OH radicals

**Fig. 2.**
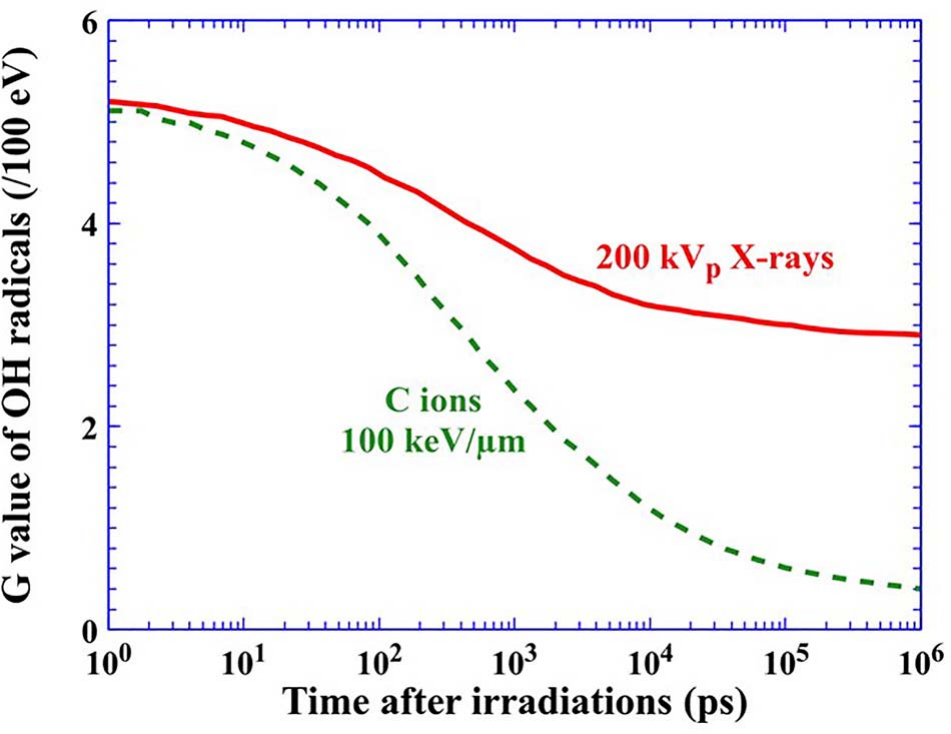
G values of OH radicals produced by 200 kVp X-rays and C ions at a LET of 100 keV/μm over time after the irradiation. The G values of OH radicals produced are simulated using a Monte Carlo simulation in Geant4-DNA.

Radiation-induced OH radicals are formed within a few picoseconds after irradiation [29]. Some of the OH radicals react with DNA molecules, but others recombine with each other or other water radiolysis products, so-called radical–radical reaction. There are also many OH radicals that diffuse without reactions in the early physicochemical stage. As time passes after the irradiation, we can see a reduction in the radiation chemical yields (G value), one entity of which is formed or destroyed for a unit of energy (traditionally 100 eV), (Fig. 2). In the case of C ions at a LET of 100 keV/μm (dotted line), the G value decreases more rapidly than that of 200 kVp X-rays (solid line) [30].

**Fig. 3.**
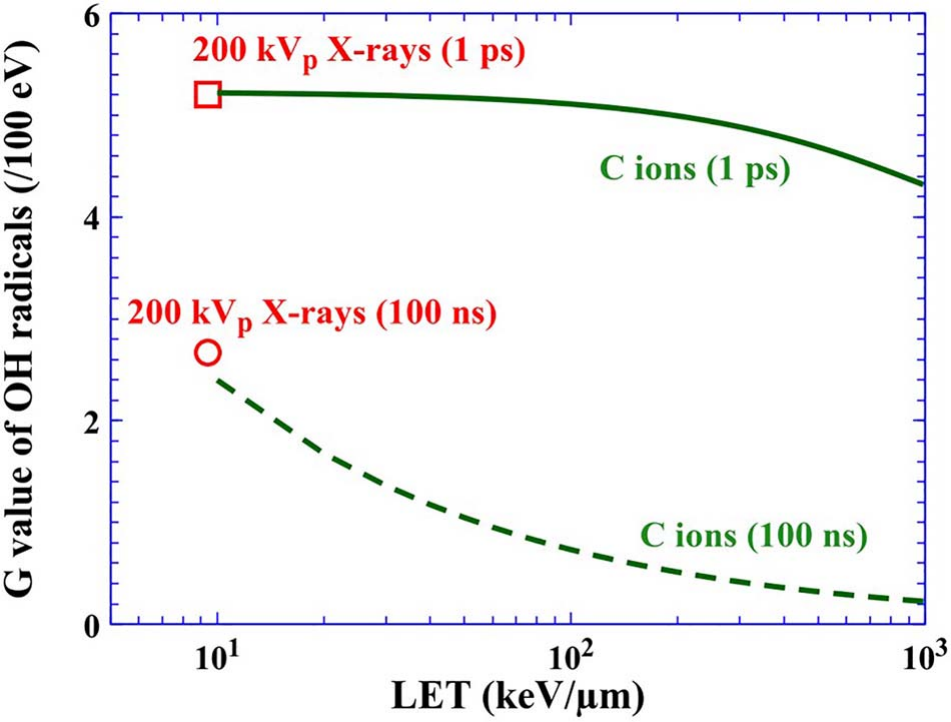
Changes in G values of OH radicals produced by C ions 1 ps (solid green line) and 100 ns (dotted green line) after the irradiation as a function of LET. The G values of OH radicals produced 1 ps (squares) and 100 ns (circles) after irradiation with 200 kVp X-rays are also shown.

Figure 3 shows G values of OH radicals produced 1 ps (solid line in green) and 100 ns (dotted line in green) after the irradiation of C ions as a function of LET. In this figure, G values of 200 kVp X-rays 1 ps (red open square) and 100 ns (red open circle) are also represented [31]. The effective energy of 200 kVp X-rays and LET are ~83 keV and 9.4 keV/μm, respectively [32]. The G value decreases monotonically with increasing LET. A dense continuous column of reactive species, which is made up of a spherical separate cluster of reactive species, a so-called ‘spur’, can be expected with increasing LET. Therefore, such radical–radical reactions are more prominent with increasing LET [30]. Therefore, 100 ns after the irradiation at which radical–radical reaction subsides, we can see a strong LET dependence of G value of OH radicals produced compared to that 1 ps after the irradiation.

### Conventional survival curve and derivation of RBE

**Fig. 4.**
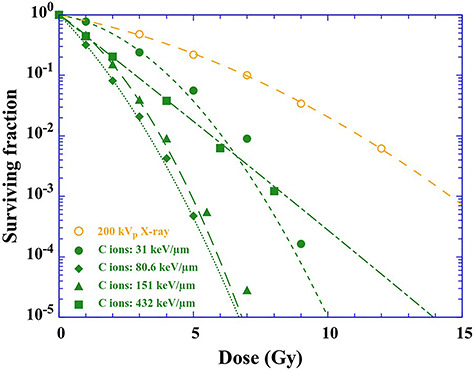
Surviving fraction of 200 kVp X-rays and C ions as a function of the absorbed dose. Data of 200 kVp X-rays are reproduced from Furusawa *et al.* [6]. The fitting lines are obtained by the LQ model SF_t_ = exp(−α*_D_D*−β*_D_D*^2^).

Survival curves of Chinese hamster cells (V79 cells) exposed to 200 kVp X-rays (circles) and C ions (circles, diamonds, triangles and squares) were reproduced from the literature (Fig. 4) [6]. The surviving fraction of C ions decreases rapidly with increasing LET in the range from 22.5 to 140 keV/μm [6]. After that, the surviving fraction with a LET of 432 keV/μm decreases more gently compared to that with 142 keV/μm. Furthermore, the ‘shoulder’ disappears with increasing LET. It is well known that the contribution of indirect action is much smaller than that of direct action in a high LET region. The absorption of radiation energy arises from excitation and ionization along an ion trajectory. In a DNA aqueous environment, biological damage is induced by direct interaction between charged particles and DNA molecules (direct action) or is triggered via free radicals (indirect action). In the latter case, the energy absorption could occur in water molecules and the free radicals produced diffuse to DNA molecules. So, even if the contribution of the shoulder is gone, it does not mean that the role of indirect action has disappeared in a high LET region [33].

**Fig. 5.**
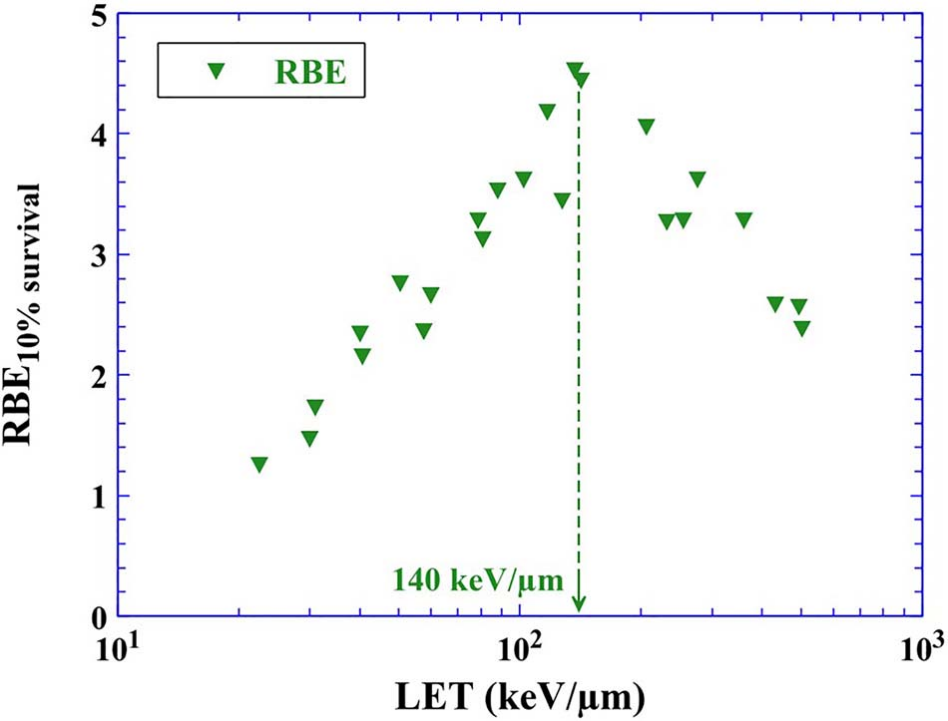
Distribution RBE at 10% survival level for C ions as a function of LET [6]. All data are the ratio to survival after exposure to 200 kVp X-rays.

Conventionally, RBE has been evaluated as the ratio of D_10_ value (absorbed dose at which the surviving fraction is 0.1) of one type of ionizing radiation relative to another (generally X-rays). From D_10_ values obtained from the LQ model, we evaluate RBE_10% survival_ for C ions (Fig. 5). Values of RBE_10% survival_ increase monotonically with increasing LET up to 140 keV/μm and then drop [6]. In the case of C ions, the value of RBE significantly rises and varies much more for different physical and biological parameters [34], compared to protons. This finding is related to the effect of radiation quality. For this reason, a more detailed model is required for heavy ions [35].

**Fig. 6.**
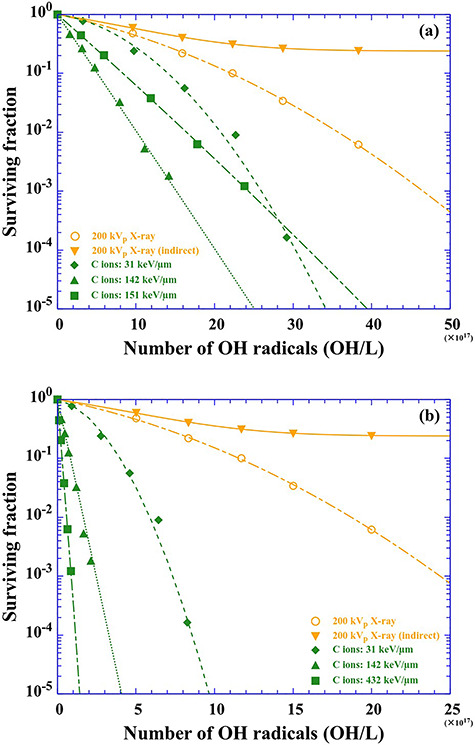
Surviving fraction of 200 kVp X-rays and C ions as a function of the number of OH radicals produced at (**a**) 1 ps and (**b**) 100 ns after the irradiation. The number of OH radicals produced is derived from G value and absorbed dose. The fitting lines are obtained by the LQ model as a function of the number of OH radicals produced SF_t_ = exp(−α*_OH_N*−β*_OH_N*^2^).

### Description of survival curves using the number of OH radicals produced

We describe survival curves of 200 kVp X-rays (circles) and C ions (diamonds, triangles and squares) as a function of the number of OH radicals produced 1 ps after ion passes, instead of the absorbed dose (Fig. 6a). The survival curve of indirect action of 200 kVp X-rays are also plotted (downward triangles). The fitting curves in Fig. 6 are derived from the LQ model. The survival curves are properly fitted by the LQ model even when they are not expressed by the absorbed dose. In addition, since the contribution of direct action is ignored, the fitting curves approach a certain value, but they never become 0. The trend of the survival curves expressed by the number of OH radicals produced is in agreement with those described by the absorbed dose. The model was developed from microdosimetric principles and equates the cellular surviving fraction to the product of two exponential terms, which depends on the first and second power of the dose as indicated in equation (1) [36]. Chadwic and Leenhouts expressed this equation from simple assumptions based on the molecular nature of the lethal event [37]. In accordance with this assumption, double-strand breaks in cellular DNA should result in cell lethality. The double-strand breaks are considered as single events as well as arising from two independently generated single-strand breaks in opposite DNA strands (double-events). When a double-event is dominant, the rate of induction of single-strand breaks (often induced by indirect action) in cellular DNA would be related to the rate of lethality of cells. In the present study, we describe survival curves of V79 cells by the number of OH radicals produced and obtain best-fit values of α*_OH_* and β*_OH_*. Clustered DNA damage often occurs along ion traversal, around which high ionization density can be expected relative to the track penumbra region [38]. Clustered DNA damage leading to cell killing is more easily induced by direct action compared to indirect action [16, 39]. Of course, damage of DNA by indirect action induced by OH radicals would also occur. However, the types of damage by indirect action (e.g. single-strand break or base mismatch) are easily and quickly reparable, except for the rare case of ‘clustered OH radicals’ occurring near the DNA (i.e. in the hydration shell with a distance of ~1 nm). The DNA damage induced by ‘clustered OH radicals’ is considered as a single-event and is taken into account as a part of direct action because its time scale is ~1 ns [18]. Thus, cell killing results from multiple hits from a single densely ionizing track from any combination of radiolysis products (e.g. OH radicals and e^−^_aq_) and ionized or excited DNA molecules (e.g. DNA^·+^ and DNA^·−^). Radiation-induced radicals play roles not only in indirect action but also in direct action. This view implies the validity of the description of survival curves using the number of OH radicals produced, instead of absorbed dose.

To follow the role of OH radicals in the chemical phase, we describe the survival curve using OH radicals produced 100 ns after the irradiation, at which time the radical–radical reaction subsides (Fig. 6b). The surviving fraction decreases rapidly with increasing LET. This trend implies that indirect action is not likely to contribute to the cell killing. The G values simulated using Geant4-DNA is in agreement with that evaluated using radical scavengers (e.g. coumarin-3-carboxylic acid (C3CA) solution) [23]. So, we would consider that the number of OH radicals scavenged by C3CA is equivalent to that attacking DNA molecules. In other words, the contribution of indirect action is equal at the same abscissa point. We emphasize here that the radiation quality is more properly taken into account using the number of OH radicals produced.

### A new concept DABBLE

**Fig. 7.**
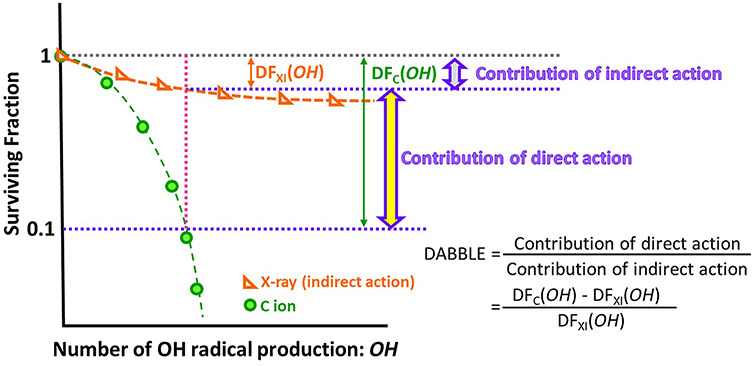
Schematic view of the derivation process of DABBLE.

As mentioned above, we adopt the number of OH radicals produced for the survival curves of V79 cell irradiated with C ions. Previously, it was demonstrated that the number of OH radicals produced could be treated as a universal criterion for evaluating the influence of radiations, while considering radiation quality [13, 14]. So, once again, the contribution of indirect action is equal at the same abscissa point. A schematic diagram of the derivation process of DABBLE is sketched in Fig. 7. To derive DABBLE, we first pay attention to the number of OH radicals produced when the surviving fraction is 0.1, namely the death fraction of cells irradiated with C ions, DF_C_(*N_OH_*), is 0.9 (green arrow). At the same abscissa point, the death fraction of cells due to indirect action by X-rays, DF_XI_(*N_OH_*) (orange arrow), is calculated from equation (7). Once again, this DF_XI_(*N_OH_*) is equivalent to the death fraction due to indirect action induced by C ions, so that the thick purple arrow in Fig. 7 represents the contribution to indirect action by C ions. Therefore, DF_C_(*N_OH_*) − DF_XI_(*N_OH_*) (thick yellow arrow) indicates the contribution of direct action induced by C ions. From this view point, we propose DABBLE as,

(8) \begin{equation*} \mathrm{DABBLE}=\frac{{\mathrm{DF}}_{\mathrm{C}}\left({N}_{OH}\right)-{\mathrm{DF}}_{\mathrm{XI}}\left({N}_{OH}\right)\ }{{\mathrm{DF}}_{\mathrm{XI}}\left({N}_{OH}\right)} \end{equation*}

The denominator expresses the death fraction due to indirect action by X-rays and the numerator indicates the death fraction due to direct action by C ions. Namely, DABBLE indicates the ratio of direct action to indirect action (Fig. 6).

**Fig. 8.**
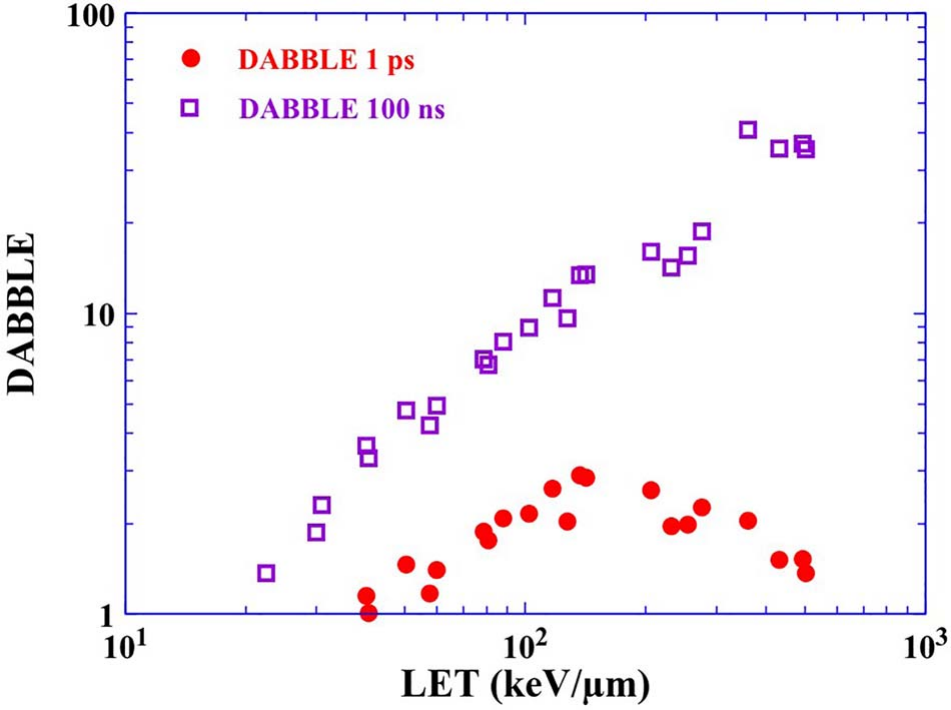
Distribution of DABBLE at 10% survival of cells after C ion irradiation as a function of LET of C ions. All data are obtained from DABBLE = {DF_C_(*N_OH_*)−DF_XI_(*N_OH_*)/ {DF_XI_(*N_OH_*)}, where DF_C_(*N_OH_*) is always 0.9. The circles indicate the DABBLE obtained by the survival curve described by the number of OH radicals produced 1 ps after irradiation and the squares represent that 100 ns after irradiation.

Fig. 8 indicates the distribution of DABBLE for C ions as a function of LET. The value of DABBLE increases monotonically with increasing LET up to 140 keV/μm, when the survival curves are described by the number of OH radicals produced 1 ps after ion pass (circles). Then, the value of DABBLE drops. This trend is in agreement with that of RBE_10% survival_, i.e. the contribution of direct action and quasi-direct action increases with increasing LET up to 140 keV/μm. The distribution of DABBLE agrees with previously reported RBE of direct action obtained from experiments using dimethylsulfoxide (DMSO) [28]. Above 140 keV/μm, the value of DABBLE decreases with increasing LET. In a high LET region, high ionizing density can be expected along the ion path. DABBLE (and RBE) is lowered because a sufficient amount of energy is deposited to cell killing above 140 keV/μm. Damage to DNA molecules induced by indirect action would be reparable. In other words, not all deposited energy contributes to cell killing. As mentioned above, water radiolysis products contribute not only to indirect action but also to direct action. Therefore, the distribution of DABBLE–LET is similar to RBE–LET. Focusing on the difference in the vertical axis, we can more easily estimate the contribution of direct action. Also, the value of DABBLE increases monotonically with increasing LET, when the survival curves are expressed by the number of OH radicals produced 100 ns after ion pass (squares). At the middle of the chemical phase (100 ns), damage to DNA molecules by OH radicals is assigned to indirect action because direct ionization or excitation leading to cell killing is gone. DABBLE expresses the ratio of direct action to indirect action. The value of DABBLE increases monotonically with increasing LET, i.e. the ratio of direct action increases relative to indirect action, which implies that the contribution of indirect action is not dominant for cell killing. Dense water radiolysis products can be expected along trajectories of heavy ions in the early stage of the chemical phase. In such cases, water radiolysis products diminish as a result of intra-track reaction, for instance, radical–radical reaction and reactions of water radiolysis products with DNA molecules, which could induce DNA damage (i.e. quasi-direct action or indirect action). However, in the middle of the chemical phase (e.g. 100 ns) the distribution of water radiolysis products is sparse relative to that at the early stage of the chemical phase. Thus, DNA damage induced by ‘clustered OH radicals’ is hardly seen. Similarly, the G value of OH radicals for X-rays does not decreases rapidly compared to that for C ions. So, the distribution of water radiolysis products in X-ray exposures would be sparse, meaning that DNA damage induced by ‘clustered OH radicals’ is not likely to occur. If cell killing by indirect action induced by OH radicals is dominant, the value of DABBLE does not increase monotonically with LET. In other words, indirect action does not act effectively with C ion therapy. Therefore, we can say that the effectiveness of C ion therapy is strongly relevant to direct action. Based on DABBLE, we can follow the contribution of indirect action over time in the chemical phase.

Direct measurement or approximation of induced DNA damage complexity along ion tracks still has many technical difficulties to overcome. DABBLE has a strong correlation with the lethality and the complexity of DNA damage. In fact, the contribution of water radiolysis products to biological effectiveness has been actively investigated. However, the toxicity of radical scavengers mixed with cells and the time dependence of roles played by water radiolysis products has not been properly elucidated. There is strong evidence, based on chemical data, that DNA base degradation products are formed by the attack of OH radicals [40, 41]. This finding implies that the contribution of OH radicals is crucial to elucidate the DNA damage leading to cell killing. Focusing on the number of OH radicals produced and the difference of surviving fraction of cells, the contribution of direct action (quasi-direct action) can be simply evaluated, while considering the difference in the radiation quality. The novel concept of DABBLE is very useful in the radiation mixed field (i.e. accelerator-based neutron field) [42, 43]. In a further step, we should consider the dependence of the cell cycle [44]. Generally speaking, the influence of the cell cycle on the surviving fraction is significant in a low LET region. This implies that the dependence on cell cycle could be important in a low DABBLE region. The V79 survival curve data used in this study were of an asynchronized cell population in the exponential growth phase [6]. Therefore, the radiation sensitivity of the cell cycle phase cannot be directly estimated from our study. An improved model considering the cell cycle should be developed to comprehensively describe the surviving fraction.

## CONCLUSIONS

We investigated the contribution of direct action relative to indirect action using our new concept DABBLE. When the survival curves were described by the number of OH radicals produced 1 ps after ion pass, the value of DABBLE increased monotonically with increasing LET up to 140 keV/μm and then dropped. This trend was in agreement with that of RBE_10% survival_ and the previously obtained RBE of indirect action. In comparison to this, the value of DABBLE increased monotonically with increasing LET, when the survival curves were expressed by the number of OH radicals produced 100 ns after the irradiation. These trends demonstrated that indirect action was not likely to contribute to cell killing. Therefore, the effectiveness of C ion therapy depends of the contribution of direct action. Using the number of OH radicals produced on the horizontal axis of survival curves and focusing on the difference in the vertical axis, we can follow the contribution of direct action over time in the chemical phase.

## CONFLICT OF INTEREST

None declared.
